# New Appliances and Things Medical

**Published:** 1905-02-18

**Authors:** 


### NEW APPLIANCES AND THINGS MEDICAL.

### SURGICAL SPONGES.

We have had submitted to us some particularly good samples of surgical sponges by Messrs. Cresswell Brothers and Schmitz (18 and 19 Red Lion Square, W.C.), including flat abdominal and honey-comb and fine turkey sponges. To those who hold, as most do, that there is no equal substitute for the natural article, we can strongly recommend the sponges before us. The flat abdominal sponge is the best we have seen. It is as thin as can be desired, while being of even consistence throughout and very soft. The price is moderate.

